# Complicated Infective Endocarditis Due to Methicillin-Sensitive Staphylococcus aureus and Streptococcus canis Bacteremia: A Multisystem Case Requiring Interdisciplinary Management

**DOI:** 10.7759/cureus.96614

**Published:** 2025-11-11

**Authors:** Abigail E Smith, John M West, August Anderson, Patrick Yue

**Affiliations:** 1 Department of Medicine, Medical College of Georgia, Augusta University, Augusta, USA; 2 Division of Infectious Diseases, Medical College of Georgia, Augusta University, Augusta, USA

**Keywords:** case report, infective endocarditis, methicillin-sensitive staphylococcus aureus (mssa), multidisciplinary management, streptococcus canis

## Abstract

We present a complex case of a 36-year-old female with right-sided infective endocarditis (IE) due to methicillin-sensitive *Staphylococcus aureus* (MSSA) and *Streptococcus canis* bacteremia, with the initial source attributed to a chronic toe wound exposed to canine saliva. Her course involved multiple organ systems with complications including septic pulmonary emboli, valvular vegetations, suspected septal abscess, third-degree atrioventricular block, and evidence of septic arthritis. The complexity of her case required coordinated management between infectious diseases, cardiothoracic surgery, electrophysiology, orthopedic surgery, interventional radiology, and cardiac critical care teams. Despite persistent bacteremia and being a high-risk surgical candidate, interdisciplinary collaboration enabled eventual stabilization for surgical intervention. This case highlights the diagnostic and therapeutic challenges of managing complicated, polymicrobial IE and exemplifies the crucial role of a multidisciplinary approach in treating this complex case.

## Introduction

Despite advancements in treatment, infective endocarditis (IE) continues to have a relatively high degree of morbidity and mortality. This is particularly true in cases involving *Staphylococcus aureus*, with a recent study finding the highest one-year mortality in IE cases among those with confirmed *S. aureus* or *Enterococcus* bacteremia [1]. Indeed, *S. aureus* has become the most common causative agent of IE in developed countries, with most other cases due to *Streptococcus* or *Enterococcus* species [1]. Left-sided IE represents most cases, with right-sided IE accounting for 5-10% of cases, many of which are associated with IV drug use [2].Complications of IE are numerous and may involve a wide variety of organ systems in addition to the heart itself. Cardiac complications can include valve destruction or perforation, abscess formation, and atrioventricular (AV) block [3]. In terms of extracardiac complications, right-sided IE notably has a significant incidence of septic pulmonary emboli [4].Due to the numerous associated complications and significant mortality, a collaborative, multidisciplinary approach to management has been recommended [5]. Such an approach involving a multidisciplinary team of physicians from several specialties has been shown to significantly reduce in-hospital mortality [6]. Here, we present a challenging case of right-sided IE complicated by septic pulmonary emboli, suspected interventricular septal abscess, and AV block, which required complex, coordinated management between multiple surgical and medical services.

## Case presentation

**Figure 1:**
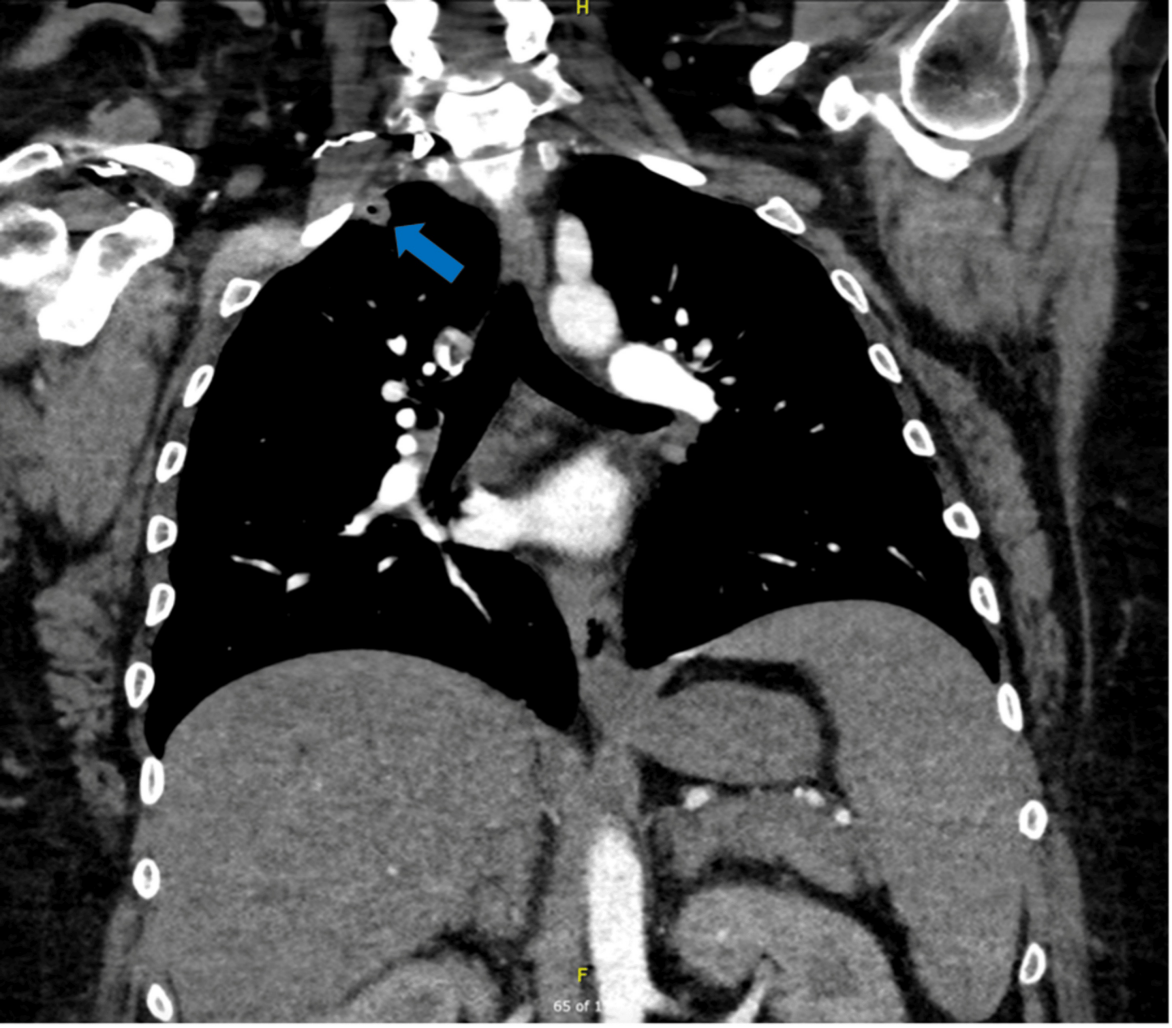
Computed tomography pulmonary angiography Coronal view demonstrating peripheral, wedge-shaped nodules concerning for septic pulmonary emboli (blue arrow).

A 36-year-old female with a past medical history of uncontrolled diabetes, asthma, eosinophilic esophagitis, and gastroesophageal reflux disease presented with midabdominal pain radiating to the chest associated with nausea, vomiting, and diarrhea of four days' duration. She also reported dyspnea and right shoulder pain. She was febrile, tachycardic, and tachypneic on arrival. The exam showed an ill-appearing female in mild respiratory distress, tachycardia, and a right great toe ulcer. Laboratory findings revealed a leukocytosis of 16.7 x 10^9^/L, as well as elevated and up-trending troponin levels. CT angiography of the chest demonstrated scattered wedge-shaped peripheral nodules suggestive of septic pulmonary emboli (see Figure 1). She met sepsis criteria and was subsequently admitted to the intermediate care unit for concern for IE. Empiric antibiotic therapy with vancomycin and ceftriaxone was initiated.

**Figure 2:**
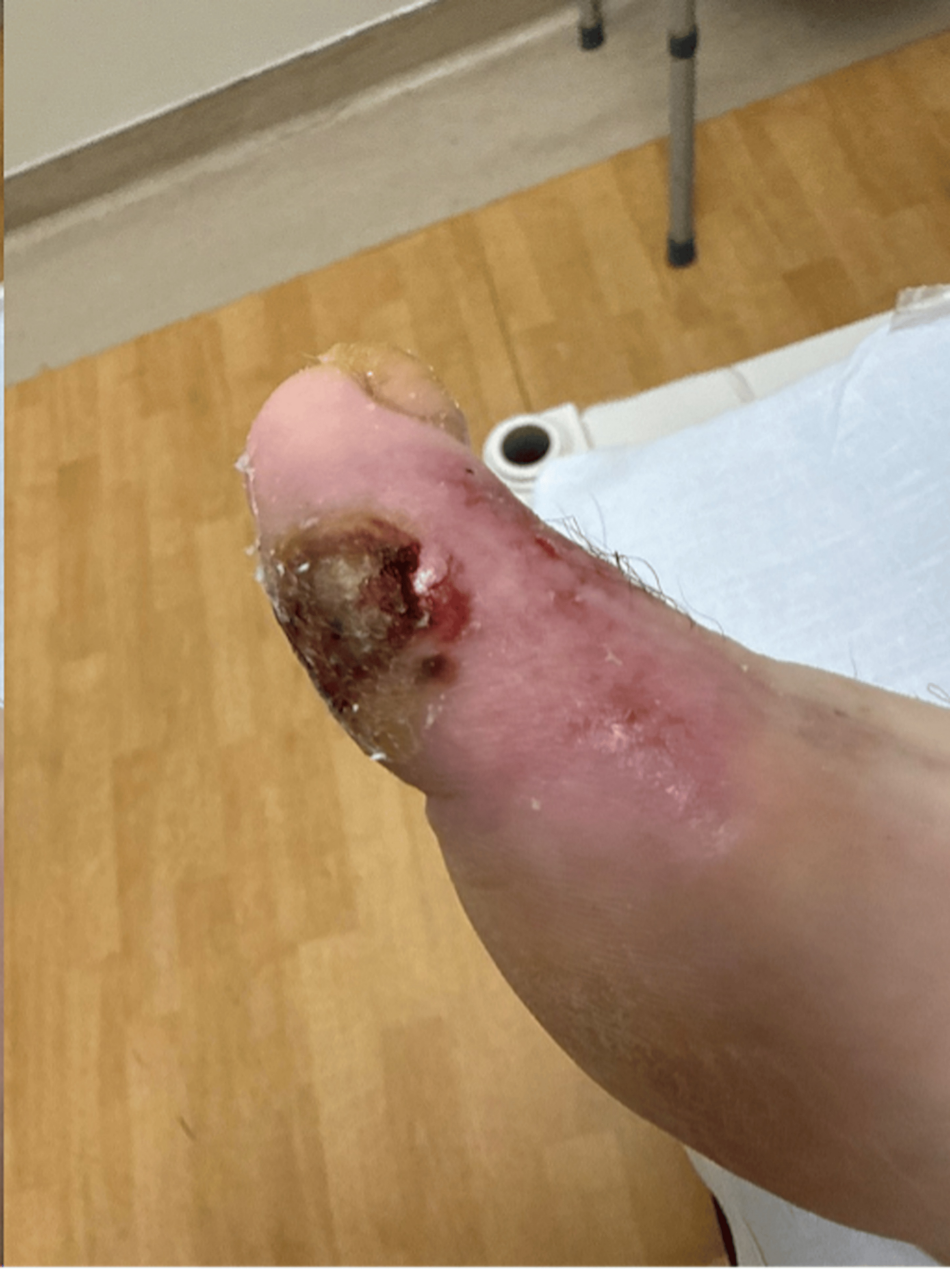
Clinical image of the chronic wound The image shows a chronic, non-healing wound about the right great toe at the time of initial presentation.

**Figure 3:**
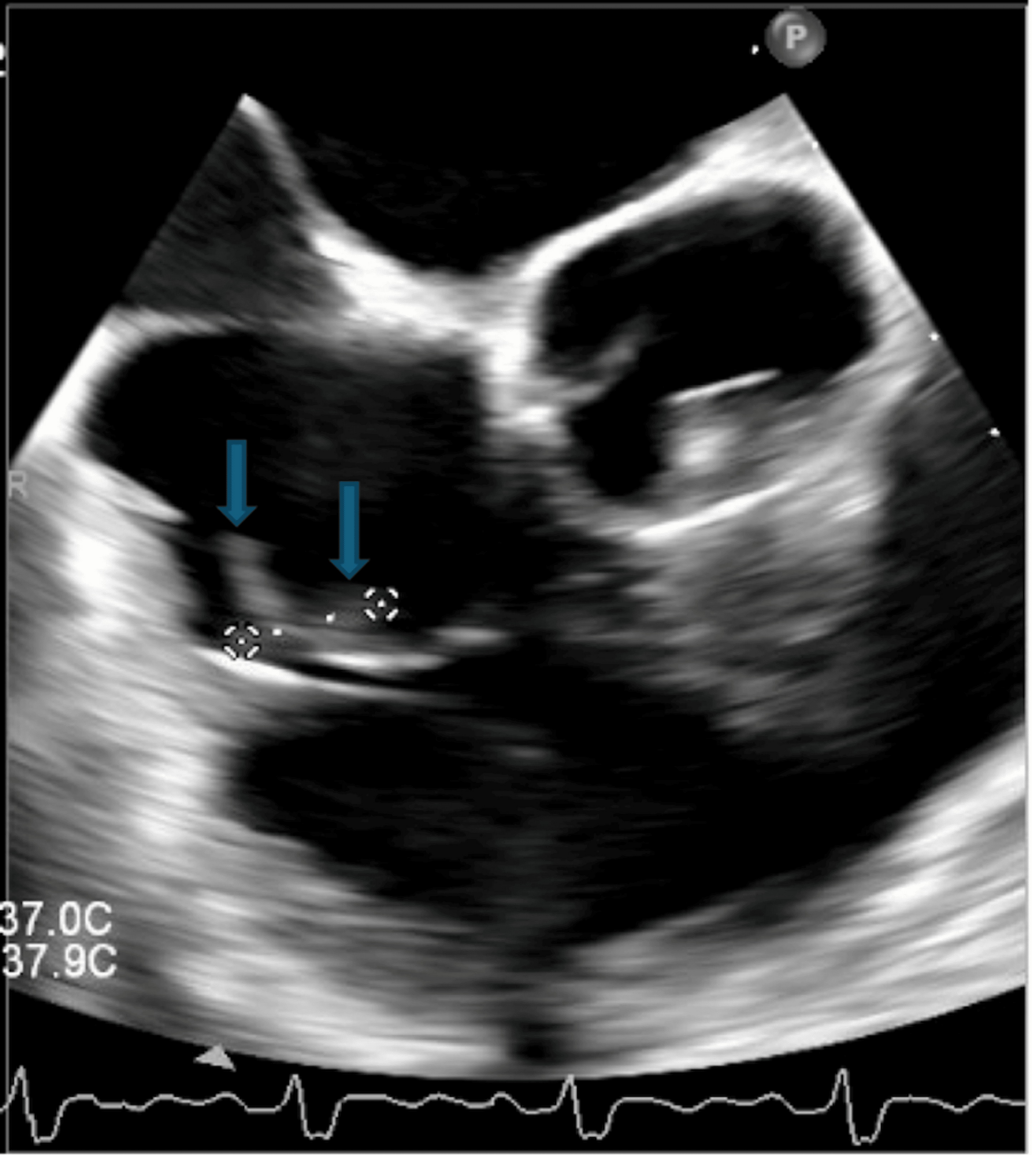
Transesophageal echocardiogram The image demonstrates a large vegetation measuring 1.32 x 1.35 cm adherent to the tricuspid valve (blue arrows).

Blood cultures collected initially grew methicillin-sensitive *Staphylococcus aureus* (MSSA) and *Streptococcus canis*. The patient later disclosed that her dog had been licking her chronic toe wound, likely serving as the source of bacteremia (see Figure 2). The patient had no reported history of IV drug use. She was switched to cefazolin per the infectious diseases team's recommendations. The initial transesophageal echocardiogram (TEE) demonstrated a normal ejection fraction estimated to be 55-60% and a moderately-sized, mobile vegetation on the tricuspid valve with an eccentric regurgitant jet suggestive of leaflet perforation (see Figure 3). Her hospital course was further complicated by a new episode of bradycardia with third-degree AV block, requiring placement of a temporary pacemaker by electrophysiology and transfer to the cardiovascular intensive care unit. A repeat TEE was performed four days later and revealed a new decline in left ventricular systolic function with an estimated ejection fraction of 35-40%, an interval increase in the size of the tricuspid vegetation, a new small vegetation on the pulmonic valve, and a thickened, heterogeneous appearance of the interventricular septum concerning for possible abscess. These findings were highly suggestive of right-sided IE with concern for ventricular septal abscess.

**Figure 4:**
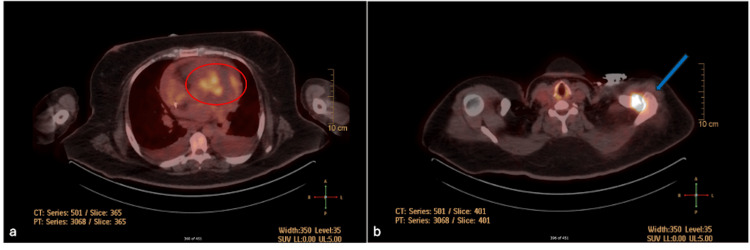
Positron emission tomography scan (a) Fluorodeoxyglucose (FDG)-avid activity in the anterior septum, mid-lateral ventricular wall, and pulmonic valve suggestive of focal endocarditis (red circle). (b) Intense FDG uptake at the level of the left acromioclavicular joint suspicious for possible septic arthritis (blue arrow).

In addition, bilateral pleural effusions found on a CT chest prompted bilateral percutaneous pigtail catheter placement by interventional radiology for empyema prevention. A cardiac MRI was indicated to further characterize the extent of the valvular vegetations; however, the temporary pacemaker prevented this due to not being MRI-compatible. In lieu of the cardiac MRI, a whole-body positron emission tomography (PET) scan was performed and showed fluorodeoxyglucose (FDG)-avid activity in the anterior septum, mid-lateral ventricular wall, pulmonic valve, and left acromioclavicular joint, suggestive of focal endocarditis and embolic septic arthritis (see Figure 4). Joint aspiration of the left shoulder by orthopedic surgery did not produce any fluid, but septic arthritis could not be ruled out. Following new pain and effusion in the left knee, joint aspiration was performed by orthopedics, with the joint aspirate analysis showing negative cultures. Cardiothoracic surgery followed the case throughout her hospital course and elected to defer tricuspid valve replacement and septal abscess debridement due to high-risk stratification in the setting of persistent bacteremia and a pacemaker.

Blood cultures remained persistently positive with MSSA despite appropriate antibiotic treatment with cefazolin, likely secondary to unresolved septal abscess. On the 12th day of admission, ertapenem was added, as limited evidence showed it has a synergistic effect with cefazolin. After day 13 of antibiotic treatment, blood cultures were negative. This case was discussed extensively in an interdisciplinary conference where the need for urgent tricuspid valve replacement and septal abscess debridement was unanimously recognized. Following stabilization and two consecutive negative blood cultures, discussions ultimately led to her transfer to an outside hospital for definitive surgical intervention.

## Discussion

This case underscores several teaching points. First, this case represents a rare instance of polymicrobial endocarditis involving *S. canis*, a pathogen rarely reported as a cause of bacteremia in humans. Second, the patient’s prolonged bacteremia with multiple metastatic complications reflects a challenge in infection control when surgery is contraindicated. Lastly, this case emphasizes the critical role of multidisciplinary coordination, which ultimately allowed the patient to receive surgical intervention.

Upon the literature review, *S. canis* rarely causes bacteremia in immunocompetent patients; however, when this does happen, it is often associated with metastatic complications. Fortunately, *S. canis* is typically susceptible to narrow-spectrum antibiotics as demonstrated in this case with rapid clearance following appropriate therapy [7]. Given this rapid clearance of *S. canis*, yet the prolonged *S. aureus* bacteremia, it is likely that *S. canis* played a very minor pathogenic role in this patient's case of endocarditis. Nevertheless, its presence is notable for its rarity and interesting correlation to the patient's history of exposure to canine saliva. Due to persistent MSSA bacteremia despite cefazolin therapy, ertapenem was added for a potential synergistic effect. Dual β-lactam therapy targets complementary penicillin-binding proteins, enhancing bactericidal activity in high inoculum infections. Case series and *in vitro* studies have shown rapid clearance of refractory MSSA bacteremia, including endocarditis, with this approach [8,9]. While the data remain limited, our patient’s improvement supports considering this regimen when surgical source control is not immediately feasible. Finally, successful management in this case was made possible through close coordination among multiple specialties. Infectious disease specialists were vital in recommending organism-directed antibiotic therapy. Cardiologists and intensivists managed her heart block and hemodynamics, while cardiothoracic surgeons evaluated but initially deferred operative intervention. Orthopedics, radiology, and pulmonology played crucial roles in evaluating septic emboli and effusions. Only through consistent interdisciplinary collaboration was the patient ultimately stabilized for transfer to a surgical center.

## Conclusions

A detailed history and physical exam can prompt identification of zoonotic risk for complicated right-sided IE, as in this patient with bacteremia involving the unusual organism of *S. canis*. In addition, early initiation of diagnostic studies and timely involvement of relevant specialties are critical for the effective management of this condition that carries a high morbidity and mortality. Our successful treatment using dual antibiotic therapy with cefazolin and ertapenem due to the reported synergistic effect supports the usage of this regimen in refractory cases with persistent bacteremia. However, the data for this synergistic effect remain quite limited, and further research is necessary to better elucidate the efficacy of such an approach. The involvement of a multidisciplinary team has substantial evidence in the literature that it improves clinical outcomes and was essential to achieving a positive outcome for this patient. The early collaboration of a team of relevant specialists should be pursued in cases of IE, particularly in highly complex cases such as this, involving the development of multiple complications.
